# Contribution of volume overload to the arterial stiffness of hemodialysis patients

**DOI:** 10.1080/0886022X.2017.1279552

**Published:** 2017-01-24

**Authors:** Łukasz Czyżewski, Janusz Wyzgał, Emilia Czyżewska, Janusz Sierdziński, Łukasz Szarpak

**Affiliations:** a Department of Nephrology Nursing, Medical University of Warsaw, Warsaw, Poland;; b Department of Laboratory Diagnostics, Medical University of Warsaw, Warsaw, Poland;; c Department of Medical Informatics and Telemedicine, Medical University of Warsaw, Warsaw, Poland;; d Department of Emergency Medicine, Medical University of Warsaw, Warsaw, Poland

**Keywords:** Arterial stiffness, electric impedance, dialysis, cardiovascular diseases, pulse wave velocity

## Abstract

Arterial stiffness is evaluated with the measurement of pulse wave velocity (PWV), while overhydration (OH) and nutritional status are evaluated with bioimpedance spectroscopy (BIS). In this study, we investigated the effect of a single dialysis session on arterial stiffness, hydration status, and laboratory parameters. The observational, cross-sectional, cohort study included 71 HD patients with mean age 64 ± 16 yrs. A Complior device was used to perform PWV measurements. The patients were examined immediately before and 15 min after a mid-week hemodialysis session. Body fluids and nutritional status were studied using a Body Composition Monitor (BCM), Fresenius Medical Care. Clinical and laboratory data were also analyzed. Multivariate regression analysis of PWV before HD showed that an OH increase of 1 L relate to a PWV parameter rise before HD of 0.523 m/s. Multivariate regression analysis of PWV after HD showed that a rise of central SBP after HD of 10 mmHg relate to a PWV increase after HD of 0.707 m/s. Our data indicate that hydration status and blood pressure may be major determinants of PWV in HD patients.

## Introduction

Cardiovascular diseases (CVD) are the main cause of death (40% of mortality) among end-stage renal disease (ESRD) patients,^1^^,^^2^ presumably because of advanced atherosclerosis. Arterial stiffness is evaluated with the measurement of pulse wave velocity (PWV),^3^^,^^4^ while overhydration (OH) and nutritional status are evaluated with bioimpedance spectroscopy (BIS). Volume overload may constitute another CVD risk in chronic hemodialysis (HD) patients, along with age, hypertension, and atherosclerosis.^5^^,^^6^ There are several studies on the subject with similar parameters being investigated. Results available from studies assessing the influence of the HD procedure on PWV are ambiguous, that is in some of them PWV is shown to remain unchanged,^7–9^ whereas in others HD is associated with a significant increase^10^^,^^11^ or decrease^12^ in aortic PWV. The stiffer the vessels, the higher the PWV. Physiologically, the pulse wave travels along arteries at a speed of 5–10 m/s. Studies show that irrespective of age, sex, and other risk factors, a 1 m/s increase of the PWV index is associated with an increase of 14% in adjusted CV and overall mortality.^13^

Apart from the traditional cardiovascular risk factors characteristic of the general population, HD-treated patients also manifest other factors that affect the condition of the circulatory system. The mechanisms underpinning increased arterial stiffness in ESRD patients are not fully defined, but major contributors identified include vascular calcification, chronic volume overload, chronic microinflammation, lipid peroxidation, oxidant stress, sympathetic overactivity, and activation of the renin–angiotensin system.^14^^,^^15^ The most obvious clinical consequence of arterial stiffness is arterial hypertension, in particular, high pulse pressure, coronary heart disease, myocardial hypertrophy, and heart failure. Identification of patients at high risk of CVD and requiring preventive and interventional strategies is an essential step in managing HD patients. It is known that high blood pressure variability, in particular intra-dialysis hypotension during HD, has a significant impact on the risk of CVD complication. The occurrence of intra-dialysis hypotension is usually associated with excessively high body mass growth between dialyses caused by increased ultrafiltration and/or too low specified dry body mass.^16^

In this study, we investigated the effect of a single dialysis session on arterial stiffness, hydration status, and laboratory parameters.

## Materials and methods

### Study population

The observational, cohort study was carried out in May–July 2015. The 71 patients (mean age 64 ± 16 yrs) who qualified for the study were being hemodialyzed in the Department of Nephrology, Dialysotherapy and Internal Medicine, Medical University of Warsaw, Warsaw, Poland, and the Dialysis Unit of the Infant Jesus Teaching Hospital in Warsaw, Poland. With written and oral instructions, patients were informed about the rules, aims, and benefits of the study and gave their written consent to participation. The inclusion criteria specified that each patient should be tested prior to the midweek HD session, have a stable clinical status, receive standard HD with three dialysis sessions per week, be on HD for at least 3 months prior to study enrollment, be free of overt CVD, and be more than 18 years of age. Excluded from the study were patients who: had atrial fibrillation, since this condition makes pulse wave velocity testing uninterpretable; had an active inflammatory process; had peripheral artery disease; had stage III–IV congestive heart failure according to the New York Heart Association classification; had regular cardiovascular instability on dialysis (dialysis hypotension for >5% of dialysis sessions over the preceding 6 months); were pregnant; had residual diuresis >500 mL/24 h; had active arteriovenous fistulas on both upper extremities; had metallic implants (stents, pacemakers, etc.); and had undergone amputation of lower or upper limbs.

### Study design

The study protocol was approved by the local Research Ethics Committee at the Medical University of Warsaw (approval number: KB/70/2015) and registered at the Clinical Trials register (www.clinicaltrials.gov, identifier NCT02443376). The investigation conformed to the principles outlined in the Declaration of Helsinki. PWV and BIS measurements were performed during midweek HD sessions by a trained researcher. Body mass, height, waist circumference, and body mass index (BMI) were assessed. BMI was calculated as body weight (kg) divided by the square of body height (m). Abdominal obesity was defined as a waist circumference of 102 cm in men and 88 cm in women.^3^

The results of laboratory investigations [C-reactive protein (CRP), haemoglobine (Hb), red blood cell distribution width (RDW), calcium (Ca), phosphate (P), calcium–phosphate index (CaxP), urea reduction ratio (URR), parathyroid hormone (PTH), total cholesterol, HDL and LDL cholesterol, and triglyceride)]were measured on the same day as PWV and BIS measurements and were obtained from the patient’s medical records. All laboratory parameters were measured using automated and standardized methods.

### Brachial blood pressure measurement

Brachial blood pressure was recorded in the dominant or non-fistula arm using a validated oscillometric device (BR-102 plus Schiller AG, Baar, Switzerland), recommended by the *European Society of Hypertension* (ESH). This measurement allowed brachial systolic blood pressure (bSBP) and brachial diastolic blood pressure (bDBP) to be obtained. The values were reported as the mean of two stable readings.

### Pulse wave analysis and central blood pressure assessment

PWV measurements were performed with the use of a Complior device (Artech Medical, Pantin, France). Patients were examined in a quiet, air-conditioned room after a 15-min rest in a supine position, directly prior to and 15 min after the commencement of the mid-week HD session. In brief, one of the device’s two sensors was placed on the site of palpable pulse on the carotid artery, and the other on the site of palpable pulse on the femoral artery. The time (*t*) between the appearance of the pulse wave on the carotid and femoral artery was measured automatically in 10 subsequent cycles (successive beats) and was averaged. Once the result of the measurement of the distance between the sensors (*m*) was complete, PWV was calculated by the device and expressed in (m/s) according to the equation: PWV = *m/t*. According to recent recommendation, when measuring PWV the distance between the sensors was automatically multiplied by coefficient 0.8 by software Complior.^17^ A higher PWV positively correlates with increased arterial stiffness.^18^ Based on brachial BP and PWV, the Complior device was also used to assess central systolic blood pressure (central SBP), central diastolic blood pressure (central DBP), as well as central pulse pressure (central PP). Intra- and intersession variability of PWV, central SBP, and central DBP obtained during reproducibility studies were acceptable (<5%).

### Hydration status assessment

The study of body fluids and nutritional status was performed using a bioimpedance monitor (Fresenius Medical Care, Bod Hamburg, Germany (BCM) Body Composition Monitor software version: 3.3.0.1637) that measures 50 different frequencies ranging from 5 to 1000 kHz, thereby analyzing the whole-body bioimpedance. The BCM is commercially available modern device that can classify ESRD subjects in terms of volume status. The bioimpedance measurement was performed directly before the commencement of HD, with the patient in a supine position and with the use of single-use disposable electrodes placed in doubles on the subject’s wrist and ankle on the side opposite to the arteriovenous fistula. The BCM discriminates fluid of the intracellular (ICW) and extracellular (ECW) water content of lean tissue mass (LTM) and overhydration (OH). LTM and OH are obtained from measurements of body weight, height, and whole-body ICW and ECW determined by BIS. OH represents the excess fluid (fluid overload). The following parameters were recorded in each patient: Lean Tissue Index (LTI (kg/m^2^)), Fat Tissue Index (FTI (kg/m^2^)), Total Body Water (TBW (L)), ECW (L), ICW (L), and OH (L). Two volume ratios were quantified: ECW/ICW and ECW/TBW.^19^^,^^20^

### Statistical analysis

Results concerning quantitative variables were presented as average values ± standard deviation (SD). In the comparative characteristics of PWV, the *T*-student test related variables were used. Univariate regression analyzes was applied to detect and describe the strength and direction of correlations of PWV to clinical, laboratory, body composition data. PWV was applied before and after HD as a dependable variable in the multivariate regression analysis. Qualitative variables (age) were presented as quantity (*n*) and percentage values of the whole group (%), while proportions in groups were assessed with the Chi-squared test. Statistica 12 software (StatSoft Inc., Tulsa, OK) was used in the statistical analysis. *p* < 0.05 was adopted as the significance level.

## Results

Among the causes of ESRD, the most frequent was glomerulonephritis (*n* = 27; 38%), followed by diabetes mellitus (*n* = 9; 13%), hypertensive nephropathy (*n* = 13; 18%), polycystic kidney disease (*n* = 11; 15.5%), and other causes (*n* = 11; 15.5%). There were 36 male and 35 female patients. The mean HD time in these patients was 84 ± 71 months. All patients underwent regular dialysis for 4–5 h and were dialyzed with standard bicarbonate HD solutions and synthetic dialyzers with blood flow rates of 200–250 mL/min, aiming at a dialysis dose of *Kt*/*V* > 1.2 with mean URR 74.1 ± 4.9%. Mean ultrafiltration (UF) during HD was 2245 ± 659 mL and bodyweight decreased by a mean of 2.3 ± 0.9 kg.

As shown in Table 1, body weight was significantly reduced in dialysis sessions. As regards arterial stiffness parameters, PWV was increased during the dialysis session. Brachial and central blood pressure remained unchanged during the dialysis session. PWV values increased during HD and statistically significant differences were registered during the dialysis session in PWV, body weight, central SBP, and central PP. Where PWV fell during the HD procedure, statistically significant differences were revealed between the beginning and the end of the dialysis session in brachial SBP, brachial DBP, central SBP, and central DBP (see Table 2).

**Table 1.** Effect of the dialysis session on brachial and central haemodynamic parameters.

Parameter	Before HD	After HD	*p*
Body weight (kg)	72.6 ± 19.6	71.4 ± 20.1	<0.001
PWV (m/s)	7.7 ± 2.1	8.7 ± 2.5	0.002
Brachial SBP (mmHg)	142 ± 25	142 ± 23	0.945
Brachial DBP (mmHg)	78 ± 12	78 ± 12	0.699
Brachial PP (mmHg)	63 ± 22	64 ± 20	0.851
Central SBP (mmHg)	138 ± 29	137 ± 24	0.869
Central DBP (mmHg)	79 ± 12	78 ± 12	0.816
Central PP (mmHg)	59 ± 27	59 ± 21	0.923
Heart rate (bpm)	72 ± 21	72 ± 14	0.761

Continuous data are presented as mean ± SD.

Statistical significances were obtained by the *T*-student test.

DBP: diastolic blood pressure; HD: hemodialysis; PWV: pulse wave velocity; PP: pulse pressure; SBP: systolic blood pressure; SD: standard deviation.

**Table 2.** Effect of the dialysis session on brachial and central haemodynamic parameters.

	PWV increases following HD, *N* = 46			PWV decreases following, *N* = 25		
Parameter	Before HD	After HD	*p*	Before HD	After HD	*p*
PWV (m/s)	7.3 ± 2.1	9.6 ± 2.2	<0.001	8.4 ± 1.9	6.9 ± 1.9	<0.001
Body weight (kg)	71.5 ± 18	70.3 ± 18	<0.001	74.9 ± 23	73.8 ± 25	0.121
Brachial SBP (mmHg)	143 ± 27	146 ± 23	0.307	140 ± 20	134 ± 22	0.021
Brachial DBP (mmHg)	78 ± 12	80 ± 13	0.346	79 ± 11	75 ± 10	0.002
Brachial PP (mmHg)	65 ± 24	66 ± 20	0.592	61 ± 19	59 ± 20	0.412
Central SBP (mmHg)	133 ± 26	142 ± 23	0.003	147 ± 34	128 ± 23	0.033
Central DBP (mmHg)	78 ± 12	80 ± 12	0.245	79 ± 11	75 ± 10	0.003
Central PP (mmHg)	55 ± 24	62 ± 21	0.015	68 ± 32	53 ± 20	0.087
Heart rate (bpm)	72 ± 18	70 ± 17	0.447	74 ± 26	74 ± 16	0.885

Continuous data are presented as mean ± SD.

Statistical significances were obtained by the *T*-student test.

DBP: diastolic blood pressure; HD: hemodialysis; PWV: pulse wave velocity; PP: pulse pressure; SBP: systolic blood pressure; SD standard deviation.

A comparative analysis between HD patients in whom PWV increases during HD session, and those in whom PWV decreases during HD session showed statistically significant differences in: OH, ECW/ICW, ECW/TBW, age, RDW, total cholesterol, HDL cholesterol, and triglycerides (see Table 3).

Results of univariate regression analyzes between PWV and clinical, laboratory, body composition data were presented in Table 4.

**Table 3.** A comparative analysis between HD patients in whom PWV increases during HD session, and those in whom PWV decreases during HD session.

Parameter	PWV increases following HD	PWV decreases following HD	*p*
*N*	46 (65%)	25 (35%)	
OH (L)	2,63 ± 0.9	1.74 ± 1.1	0.002
LTI (kg/m^2^)	12.3 ± 2.9	12.9 ± 2.4	0.524
FTI (kg/m^2^)	11.8 ± 4.8	11.3 ± 5.5	0.805
TBW (L)	33.0 ± 6.6	34.4 ± 8.9	0.626
ECW (L)	16.9 ± 2.8	16.7 ± 4.3	0.853
ICW (L)	16.0 ± 3.9	17.7 ± 4.9	0.325
ECW/ICW	1.083 ± 0.132	0.969 ± 0.149	0.044
ECW/TBW	0.515 ± 0.030	0.489 ± 0.038	0.038
Waist circumference (cm)	100 ± 11	89 ± 20	0.205
BMI (kg/m^2^)	24.9 ± 4.6	26.5 ± 7.5	0.337
Age (y)	67 ± 15	57 ± 15	0.023
Duration HD (mo)	86 ± 85	78 ± 72	0.765
CaxP	3.64 ± 1.25	4.10 ± 1.24	0.321
RDW (%)	15.2 ± 1.28	14.4 ± 0.9	0.019
PTH (pg/mL)	394 ± 367	393 ± 270	0.995
Hb (g/dL)	10.2 ± 1.1	10.7 ± 1.2	0.122
CRP (mg/L)	7.5 ± 5.5	6.3 ± 5.4	0.449
Total cholesterol (mg/dL)	159.5 ± 43.9	190.1 ± 53	0.022
HDL-cholesterol (mg/dL)	42.2 ± 12	50.8 ± 16	0.029
LDL-cholesterol (mg/dL)	92.1 ± 32.4	103.7 ± 39	0.237
Triglycerides (mg/dL)	122 ± 53	175 ± 106	0.017
Duration HD session (h)	4,2 ± 0.5	4.1 ± 0.5	0.469

Continuous data are presented as mean ± SD.

Statistical significances were obtained by the *T*-student test.

BMI: body mass index; CaxP: calcium–phosphate index; CRP: C-reactive protein; ECW: extracellular water; FTI: fat tissue index; Hb: hemoglobin; HD: hemodialysis; ICW: intracellular water; LTI: lean tissue index; OH: overhydration; PTH: parathyroid hormone; PWV: pulse wave velocity; RDW: red blood cell distribution width; TBW: total body water.

### Multivariate regression analysis of PWV before HD

One independent parameter was shown to have an essential influence on the dependable variable PWV: OH, *p* < 0.007. A 1 L rise in OH relate to a rise of the PWV parameter before HD of 0.523 m/s (see Table 5).

**Table 4.** Results of univariate regression analyzes between pulse wave velocity and clinical, laboratory, body composition data in hemodialysis patients.

	PWV before HD		PWV after HD	
Parameters	*r*	*p*	*r*	*p*
Age	0.192	0.146	0.457	<0.001
HD duration	−0.052	0.730	−0.022	0.883
Brachial SBP	0.203	0.124	0.459	<0.001
Brachial DBP	0.133	0.314	0.239	0.069
PP	0.153	0.246	0.395	0.002
Heart rate	0.006	0.965	−0.205	0.120
Central SBP	0.094	0.479	0.460	<0.001
Central DBP	0.133	0.314	0.230	0.080
Hemoglobin	0.078	0.568	−0.145	0.286
RDW	−0.154	0.256	0.166	0.221
Total cholesterol	0.249	0.066	−0.017	0.900
HDL-cholesterol	0.140	0.307	−0.123	0.372
LDL-cholesterol	0.197	0.149	0.105	0.447
Triglycerides	0.126	0.359	−0.172	0.209
CRP	0.177	0.215	0.246	0.082
BMI	0.011	0.999	0.081	0.985
OH	0.376	0.024	0.039	0.824
LTI	−0.224	0.190	−0.258	0.134
FTI	0.252	0.139	0.291	0.089

BMI: body mass index; CRP: C-reactive protein; DBP: diastolic blood pressure; FTI: fat tissue index; HD: hemodialysis; LTI: lean tissue index; OH: overhydration; PWV: pulse wave velocity; PP: pulse pressure; RDW: red blood cell distribution width; SBP: systolic blood pressure.

**Table 5.** Multivariate linear regression analysis of pulse wave velocity before hemodialysis.

Covariate	Effect	S.E.	95% CI	*p*	*R*^2^
OH	0.523	0.225	1.145–0.205	0.007	0.391

CI: confidence interval; OH: overhydration; SE: standard error.

### Multivariate regression analysis of PWV after HD

One independent parameter has an essential influence on the dependable variable PWV: central SBP after HD, *p* < 0.001. A rise of central SBP after HD of 10 mmHg relate to a rise of the PWV after HD of 0.707 m/s (see Table 6).

**Table 6.** Multivariate linear regression analysis of pulse wave velocity after hemodialysis.

Covariate	Effect	S.E.	95% CI	*p*	*R*^2^
Central SBP	0.707	0.017	0.043–0.115	<0.001	0.521

CI: confidence interval; SBP: systolic blood pressure; SE: standard error.

## Discussion

The present study provides new information on the relationship between nutritional status and hydration status, blood pressure, and PWV in HD patients during an intra-dialysis session. The main finding of this study was that in HD patients the PWV correlated with OH, a marker of the fluid status representing body fluid distribution. Our own study findings show that when PWV was increased following HD the mean OH was 2.63 ± 0.9 [L], while when PWV was decreased following HD the mean OH was 1.74 ± 1.1 [L] (*p* = 0.002). This testifies to a different arterial elasticity behavior in the patients studied depending on the excess fluid status and that OH is an independent risk factor in arterial distension. The treatment of hypertension and fluid overload are issues of major importance in HD patients.

In spite of its limitations, the use of brachial blood pressure is one of the main clinical indicators of the fluid status in ESRD patients. Central aortic BP is an indirect measure of arterial stiffness that provides valuable information on wave reflections. PWV changes during HD sessions are associated with changes in arterial wall elasticity and can consequently contribute to changes in arterial pressure during the HD procedure. A study on a group of patients with PWV decline during HD showed that brachial SBP, DBP, and central SBP and DBP decreased in a statistically significant way. The results of PWV measurement in the study population (7.7 m/s before HD) are quite low when compared with other studies in HD patient. Too aggressive a rate of ultrafiltration for dialysis lasting 3.5–4.0 h may result in inadequate dialysis, which in turn can lead to OH and the occurrence of volume-dependent hypertension with higher values of PWV. In the present study, the length of dialysis sessions varies on average between 4 and 5 h with low blood flow:- 200–250 mL/h [18 (25%) patients regularly underwent 5.0 h HD, 20 (28%) patients underwent 4.5 h HD, and 20 (47%) patients underwent 4.0 h HD]. In the study, after HD if central blood pressure increase, results as increased the PWV, while the decrease, decreased the PWV. PWV increased group have more OH be evocative of paradoxical hypertension. Paradoxical hypertension is defined by BP values during and at the end of the dialysis session exceeding BP values at dialysis onset. It occurs in around 10% of HD patients.^21^ The most important cause of paradoxical hypertension during UF is increased cardiac output, mediated by volume overload and can be treated by intensified UF.^22^^,^^23^

In the study by Georgianos et al.^24^ on 51 stable HD patients, PWV was evaluated before and after the first (after a 3-day inter-dialysis interval) and second (mid-week) dialysis session of the week. Augmentation index (Aix), SBP and PP at brachial artery, and central aorta were reduced. Aortic PWV remained unchanged during both dialysis sessions. For the first HD session: PWV before HD = 9.68 ± 0.3, PWV after HD = 9.78 ± 0.4 m/s (*p* = 0.739). For the second HD session: PWV before HD= 9.58 ± 0.3, PWV after HD = 9.48 ± 0.3 m/s (*p* = 0.830).

The study carried out by Su et al.^11^ showed that in spite of a significant decrease in body weight and BPs, PWV still increased significantly after HD (19 m/s) but returned to the pre-dialysis level on the next dialysis-free day (18 m/s).

In the study by Di Iorio et al.,^12^ PWV values have a weekly cyclic variation and show significant reductions during each dialysis session (15.6 ± 5.2 to 9.3 ± 2.3, 13.4 ± 4.0 to 8.7 ± 2.4, and 12.4 ± 2.6 to 9.2 ± 2.2 m/s, before and after the first, second, and third weekly dialysis sessions, respectively). The HD ultrafiltration rate correlated significantly with intra-dialysis PWV changes (*r* = 0.465; *p* < 0.001) and with after dialysis PWV values (*r* = −0.654; *p* < 0.0001). Blood pressure changes during dialysis were weakly correlated with post-dialysis PWV (*r* = −0.267; *p* < 0.05), but not with PWV changes during dialysis.

The study by Covic et al.^10^ showed that PWV was increased following an HD session, from 7.19 ± 1.88 to 7.89 ± 2.09 m/s (*p* < 0.004). In addition, an HD session significantly reduced AIx from pre-HD 27.9 ± 11.9% to 18.2 ± 18.3% (*p* < 0.05). With provocative pharmacological use of inhaled salbutamol and sublingual nitroglycerin testing, Covic et al. revealed that an HD session led to a non-significant improvement in the endothelium-dependent vasomotor function, while correcting the endothelium-independent dysfunction to a level comparable to that in a control group of essentially hypertensive patients.

Among others, RDW is a relatively new prognostic parameter in heart failure.^25^ An elevated RDW value is evidence of a great diversity of erythrocytes (anisocytosis). The prognostic value of RDW in patients with heart failure is comparable to NT-proBNP.^26^ In addition, the parameter is given in every complete blood count, making it cheap and generally available. The normal level of RDW ranges from 11.5% to 14.5%. The results of our own study revealed that the RDW indicator in the group of PWV patients increases following HD (before HD: 15.2 ± 1.28 m/s vs. after HD: 14.4 ± 0.9 m/s; *p* = 0.019). In their study, Yoon et al.^27^ reported a progressive rise in RDW being a predictor of mortality and cardiovascular events in ESRD patients, independent of the indices of anemia, nutrition, hemoglobin variability and traditional CV risks. Results from previous studies assessing the influence of the HD procedure on PWV are highly variable. Patient characteristics and other unknown or unmeasured factors may confound their relationship. Therefore, change of PWV during HD might be served as a useful predictor of other cardiovascular risk factors or outcomes.

## Limitation

This is a cross-sectional study in design and cannot provide cause-and-effect associations. The study measurements may be susceptible to biases related to the peridialytic setting of recordings. Data on wave reflection indices were not assessed. To find the most representative time point for PWV measurements during HD, our study design did not include patient evaluation on all three dialysis sessions of the week and inter-dialysis days.

## Conclusions

Our data indicate that hydration status and blood pressure may be major determinants of PWV in HD patients.
